# Supplementary material for: The use of 3D digital anatomy model improves the communication with patients presenting with prostate disease: The first experience in Senegal
Source: PLoS One. 2022 Dec 1;17(12):e0277397. doi: 10.1371/journal.pone.0277397 (PMC9714841; doi:10.1371/journal.pone.0277397)
Supplement: Attachment — Submitted filename: Response to Reviewers.docx [file pone.0277397.s003.docx]

**Response to the Reviews**

We thank the editor and the reviewers for the helpful comments. Our point-by-point responses below detail the revisions.

**Reviewer 1**

The changes addressing these comments are marked by green color in the revised manuscript.

**Comment**: The manuscript is simple but informative. Also the results are expected.

***Answer****: Thank you for this positive comment. We were indeed glad to see that our results support the hypothesis. [No editing is required to address this comment].*

**Comment**: The number of patients and physicians accessed was reasonable.

***Answer****: Thank you for this positive comment. [No editing is required to address this comment].*

**Comment**: While any image is better than no image, I would add simple anatomic figures to be more comprehensive to patients…. In our practice, simple and didactic anatomic pictures on the physician's office wall play similar role, certainly taking less time.

***Answer****: We agree, while noting that in our study, during the first part of the consultation session the physicians used some didactic anatomical pictures of their own design and drawing. Nonetheless uniformly reported favorable experience from additionally using 3D digital anatomy models. Hence in this paper we argue that the use of 3D digital models may help improve upon the quality of patient-physician communication. We emphasize this in the revised text.* ***[See in Methods / Study Design]***

**Comment**: It is a huge challenge the fact that "76.6% of physicians complained that using the computer model lengthened the consultation. This might reflect the complexity and inaccuracy of utilized model and interface (computer).

***Answer****: We agree that lengthening the consultation session is indeed a significant concern. The use of 3D digital models doubtless adds complexity which in turn adds time to the consultation. In the revised Discussion section, we propose ways to mitigate this effect, such as using a simpler digital anatomy viewing software.* ***[See in Discussion /*** ***Impact on physicians]***

**Reviewer 2**

The changes addressing these comments are marked by red color in the revised manuscript.

**Comment**: This manuscript presents the use of a 3D digital anatomy model of the prostate to help improve patient-provider communication in Senegal. While the concept is quite interesting, this study lacks depth and clarity. This manuscript could be strengthened with major revisions.

***Answer****: We agree and carried out substantial revisions to improve on the depth and clarity of the paper.* ***[See throughout the text.]***

**“Introduction”**

**Comment**: Should identify more current statistics on worldwide rates of prostate cancer deaths.

***Answer****: We agree and added the required information and references.* ***[See in Introduction]***

**Comment**: It is unclear how “sociocultural realities that render prostate disease a taboo subject” is ameliorated or even considered in the context of this intervention.

***Answer****: We agree and removed this aspect from the manuscript.*

**Comment**: It is also unclear how this intervention has the potential to impact limited knowledge of prostate disease that are often not seen until presented complicated or as advanced disease. This is not a community intervention on awareness, but rather a clinical intervention after clinical presentation.

***Answer****: We agree and make this distinction clear. We also broke down the results to referral groups. We further note in the revision that increasing the public awareness of prostate disease and improving the management of the disease (including patient-physician communication, the subject of this paper) must go hand in hand.* ***[See in Discussion / Relationship to public awareness of prostate disease]***

**Comment**: This authors must reconsider what is important to present as relevant background information for this study.

***Answer****: We agree and significantly revised the background*. ***[See in Introduction]***

**Comment**: Given the preliminary and exploratory nature of this study, this should be stated clearly upfront.

***Answer****: We agree and made clear the preliminary and exploratory nature of this study. We also changed the abstract to reflect this aspect.* ***[See in Abstract and in Introduction]***

**“Methods”**

**Comment**: Given the discussion of the 3D digital anatomy model section, it is unclear if a standard image was shown to all patients in the study, or if the image was personalized based on the individual patient case. It is unclear why there is a section on a specific patient case of a 62 year old man.

***Answer****: The MRI “image” was not personalized based on the individual patient case, and we clarify this important detail in the revision.* ***[See in Methods]****. We further note that in Senegal, there is a general lack of imaging capacity, especially of MRI. For future work, we consider creating several anatomical models, and based on a quick digital rectal examination, the physician could select the one that feels the most similar one to the prostate of the given patient.* ***[See in Discussion / Limitations / Choice of the 3D anatomical model]***

**Comment**: Under study design, authors indicate “patient first received … standard manual drawings, followed by … 3D digital…” What is the standard manual drawing based on? Is it just what the physician was able to draw themselves or a predrawn out image on a paper? More detail is needed for clarity.

**Answer**: *In their “conventional patient-physician communication”,* *the participating physicians used didactic anatomical pictures of their own design and drawing, according to their own usual practice. We did not provide a “standardized” image or drawing. We clarified this in the revision.* ***[See in Methods]***

**Comment**: The questions are quite limited in nature and should be addressed in a limitations section.

***Answer****: We agree, with stressing, as the reviewer noted, that this was a* *single-arm prospective qualitative study of preliminary and exploratory nature.* *The questionnaires were purposefully designed to be simple and straightforward, for reasons we discuss in the revised text.* ***[See in Discussion / Limitations / Choice of questionnaires]***

**Comment**: What, if any, incentives were provided to study participants – whether patients or providers?

***Answer****:* *No specific incentives were offered to study participants, neither patients nor providers. During the informed consent process, however, patients were informed about potential benefits, such as receiving more detailed information about their disease and condition and additional “face time” with their consulting physician*. *We clarified this in the revision.* ***[See in Methods]***

**“Results”**

**Comment**: A comparison/usual care group could be much more informative and insightful, along with more detailed questions to assess quality of communication, understanding, communication skills, etc., even for low literacy groups, with low response burden.

***Answer****: We agree that a two-arm study and more nuanced questionnaires could be more informative – and these we intend to pursue in follow-up work outlined in the revised Discussion section. We reemphasize that the purpose of this study was to gauge the perception and generic practicality of 3D digital anatomical models, while keeping the questionnaire unburdening for all participants.* ***[See in Discussion / Limitations / Study design and in Choice of questionnaires]***

**Comment**: Authors should be more clear that the questions assess perception/perceived point of view. For example, asking about improved level of understanding as improved or declined, is perceived and not objective as asking actual questions that assess knowledge. This should also be acknowledged in a limitations section.

***Answer****: We agree and clarified the issue in the revision, stressing that this was a single-arm prospective qualitative study of exploratory nature, we chose to gauge the perception of the participants, rather than to assess their knowledge and experience gained through using of 3D digital anatomy models. We reserved this objective for future work* ***[See in Discussion / Study Design]***

**“Discussion”**

**Comment**: Authors should rephrase discussion section and conclusions drawn to be more accurate. For example, quality was not assessed and the conclusion that quality of communication improved is not accurate. There was no comparison group to show an improvement, nor a pre/post questionnaire, for example.

***Answer****: We agree and clarified in the revision: this was a single-arm prospective qualitative study of exploratory nature, in which we did not have different study arms to compare. We detail this among the Limitations in the revised Discussion session.* ***[See in Discussion / Limitations / Study design and in Choice of questionnaires]***

**Comment**: Impact on physicians – There is also extreme over simplification of communication skills. Nothing that was presented in the intervention dealt with communication – e.g., what is actually said/communicated to patients. This 3D image is a visual “tool” that has the potential to enhance communication, but that was not measured in this study, but rather participants’ perspective on usability or utility, and even that is quite limited in the 4 questions asked of patients and providers.

***Answer****: We agree and clarify in the revision, as the reviewer correctly noted earlier, that this was a preliminary and exploratory study, in which we did not intend to dwell in a quantitative analysis of such outcomes. The reviewer identifies an important objective for follow-up work that we outlined in the revised Discussion section.* ***[See in Discussion / Limitations / Study design and in Choice of questionnaires]***

**Comment**: Statistical significance – It is unclear why there is a section on statistical significance when this study was exploratory in nature and only presented descriptive statistics. This section seems inappropriate.

***Answer****:* *We agree and revised the* statistical considerations *accordingly.* ***[See in Results /* Statistical Considerations*]***

**Comment**: The additional subheadings following the discussion section is confusing, when some appear to be appropriate for the methods section.

***Answer****: We agree and revised the subheadings accordingly. [****See in Discussion]***

**Comment**: Digital software – what is the difference between the two platforms mentioned, besides one being free? Why bring this up as an option?

**Answer**: *We intended to say that, for future work, there is now a newer and simpler free open-source digital anatomy platform that provides the same visual functionality as the software we used in this preliminary work. We clarified this in the revised Discussion section. [****See in Discussion / Choice of digital anatomy viewing software]***

**Comment**: The section on longevity of findings also seem inappropriate and inaccurate given the nature of this study.

***Answer****:* *We agree and* arranged it into a subsection on Robustness of findings over time. ***[See in Discussion / Robustness of findings over time]***

**Reviewer 3**

The changes addressing these comments are marked by blue color in the revised manuscript.

**Comment**: The manuscript represent the result of a survey on the use of a 3D digital anatomy model in urology consultation to reflect its effect on improving the communication of physician and patients. An interesting model used in this study which based on the results of the survey demonstrated an improved communication between physicians and patients. However, I do not believe the survey results are presented and discussed properly. in more detail (e.g.consider results in referral groups and).

***Answer****: We agree, and we carried out substantial revisions to address the issues raised by the reviewer, with emphasizing that this was a single-arm prospective qualitative study of preliminary and exploratory nature. We increased the depth of the presentation of Results and Discussion.* ***[See in Results and in Discussion].***

**Comment**. It is not clear if patients information such as age is collected in the survey. If it has been collected, the results should be reported and investigated in terms of any association with age groups (e.g. elderly patients might gain less benefit from the technology compared to younger patients). Otherwise, it should be mentioned that age of patients are not recorded in survey with a discussion on the reason.

***Answer****: Patient demographics was not collected in this study. We include this clarification in the revised manuscript.* ***[See in Methods / Study Design and in Discussion /*** ***Impact on patients]***

**Comment**: There is no statistical method applied to investigate the result of the survey. Authors need to apply statistical methods to add the uncertainty associated with the reported percentage (e.g. in terms of confidence intervals).

**Answer**: *We agree, and we extended the statistical analysis in the Results section with confidence intervals in both Tables 1 and 2.* ***[See in Results /*** ***Answers to the questionnaires]***

**Comment**: Chi‐square or other relevant tests should be applied to investigate the association of each questionary with available patient groups (referral group, age groups, etc). [The] authors need to report results of the questionaries in the survey by the referral groups and investigate any association between referral groups and each questionary (e.g. different treatment usually are recommended for patients with different group so it would be interesting to consider how patients’ decision has changed regarding their referral group).

***Answer****: We agree, and in the revised paper we break down the results by various referral groups and analysed the results. Chi-square test of independence was performed to analyze the relation between referral groups and different responses in the questionnaire. Bonferroni correction was applied to compensate for repeated tests. [****See in Table 3, in Results, and in Discussion /*** ***Impact on Patients].***

**Comment:** Impact on patients in discussion, it is mentioned that “A tool that facilitates the communication and understanding of prostate diseases could be beneficial in improving the quality of care and reducing healthcare costs”. However, it is not well discussed how such a tool could reduce healthcare cost, and I suggest authors to add a discussion justifying this (lines 168‐170)

***Answer****: We agree and added the required discussion.* ***[See in Introduction]***

**Comment:** They mentioned that the use of such a 3D digital anatomy model is helpful for treatment decision of high‐risk patients. It is not clear how the presented results are supporting such a statement! A clear discussion needs to be added including how the patients decisions are improved by use of such a 3D digital anatomy model.

***Answer****: We agree and in the revised paper, in Table 3, we break down the results by referral groups including the “high-risk” patient groups. Patients favored the digital model regardless of the referral group,* *the differences were much more significant in decision-making for “high-risk” patient groups who needed immediate management or treatment of the disease. [****See in Table 3 and in Discussion /*** ***Impact on Patients].***

**Comment:** Some of subsections in the discussion should be moved and presented in the Method section (i.e. Statistical significance, Choice of questionnaires and Choice of digital anatomy viewing software).

***Answer****: We agree, and we carried out substantial editorial revisions. We moved “Statistical considerations” to the Results section. At the same time, we kept in the Discussion under Limitations the subheadings on “*Choice of questionnaires” and “Choice of digital anatomy viewing software”; we deem these necessary to properly discuss limitations of the study. ***[See in Results and Discussion/Limitations]***

**Comment:** It is not clear how such a software would be available to be used in the clinical practices if required.

***Answer****: We included a brief discussion of this issue. [****See in Discussion / Limitations / Choice of Choice of digital anatomy viewing software]***
